# Broadening SARS-CoV-2 Immunity by Combining ORFV and Protein-Based Vaccines

**DOI:** 10.3390/vaccines14010064

**Published:** 2026-01-04

**Authors:** Alena Reguzova, Melanie Müller, Madeleine Fandrich, Alex Dulovic, Ralf Amann

**Affiliations:** 1 Institute of Immunology, University Hospital Tübingen, Auf der Morgenstelle 15, 72076 Tübingen, Germany; 2 NMI Natural and Medical Sciences Institute at the University of Tübingen, 72770 Reutlingen, Germany

**Keywords:** Orf Virus, viral vector, vaccines, heterologous prime-boost, SARS-CoV-2, VidPrevtyn Beta

## Abstract

Background: Emerging immune-evasive viral variants threaten the efficacy of current vaccines, underscoring the need for strategies that elicit broad and durable protection. Heterologous prime–boost regimens combining distinct vaccine platforms can enhance humoral and cellular immunity through complementary mechanisms. Methods: Using an intramuscular immunization scheme aligned with clinical vaccination practice, CD-1 mice received homologous or heterologous prime–boost regimens combining a replication-deficient Orf virus (*Parapoxvirus orf*, ORFV)-based spike vaccine (ORFV-S) with the licensed adjuvanted recombinant protein vaccine VidPrevtyn Beta. Spike-specific humoral and cellular immune responses were assessed. Results: ORFV-S alone induced potent and broad spike-specific IgG responses and achieved the strongest ACE2-binding inhibition across variants of concern. ORFV-S priming followed by VidPrevtyn Beta boosting markedly enhanced the magnitude and cross-variant breadth of antibody responses compared with homologous protein vaccination. Both homologous ORFV-S and heterologous regimens incorporating ORFV-S elicited strong CD4^+^ and CD8^+^ T-cell responses, whereas VidPrevtyn Beta alone induced only modest T-cell activity, demonstrating that ORFV-S effectively complements protein-based vaccines. Conclusions: The ORFV-S vector represents a potent vaccine platform capable of inducing broad humoral and cellular immunity. Its use in heterologous prime–boost combinations enhances both antibody magnitude and breadth beyond homologous protein vaccination, supporting its application in vaccination strategies against evolving viral pathogens.

## 1. Introduction

The continued emergence of immune-evasive viral variants threatens the long-term efficacy of existing vaccines [1]. This is particularly evident for rapidly evolving RNA viruses such as SARS-CoV-2 [2], influenza [3], and HIV [4], where antigenic drift can undermine vaccine-induced protection and necessitate frequent updates of immunogen composition. These challenges underscore the need for vaccination strategies that elicit cross-reactive and durable immune responses effective against different variants [5,6].

Heterologous prime–boost regimens, which combine immunologically distinct vaccine platforms, have emerged as a promising strategy to enhance both humoral and cellular immunity [7]. Through complementary activation of innate sensors and distinct antigen-presentation pathways, such combinations can overcome platform-specific limitations, mitigate anti-vector immunity, and broaden cross-variant protection [8]. The success of mix-and-match vaccination during the SARS-CoV-2 pandemic exemplifies the potential of this approach [9,10].

Recombinant protein-based vaccines, such as VidPrevtyn Beta (Sanofi–GSK, Paris, France; London, UK), offer high safety, scalability, and potent antibody induction when adjuvanted with AS03, a squalene-based oil-in-water emulsion [11,12]. VidPrevtyn Beta, based on the prefusion-stabilized spike of the Beta (B.1.351) variant, was the first next-generation COVID-19 vaccine approved in Europe and has shown strong immunogenicity in heterologous regimens [13]. However, protein-based vaccines generally elicit limited T cell responses and may benefit from combination with viral vectors that promote robust activation and cellular immunity [14,15].

The Orf virus (*Parapoxvirus orf*, ORFV), a member of the *Parapoxvirus* genus, represents an attractive vector platform owing to its restricted host-range, large genome capacity, and low anti-vector immunity, as recently reviewed by Helmold et al. [16]. ORFV activates the cGAS–STING pathway, inducing strong innate activation and potent humoral and cellular responses [17,18]. The multi-antigenic ORFV-based vaccine Prime-2-CoV, co-expressing the SARS-CoV-2 spike and nucleocapsid proteins of the ancestral Wuhan strain, demonstrated robust immunogenicity in animal models, absence of systemic replication in vivo, and an excellent safety profile in a first-in-human trial [19,20,21,22]. Moreover, the ORFV vector platform has been successfully applied in heterologous prime–boost regimens with both inactivated (VLA2001, Valneva, Nantes, France) and adenoviral (Jcovden, Johnson & Johnson, Leiden, The Netherlands) SARS-CoV-2 vaccines, demonstrating synergistic enhancement of immune responses in preclinical studies [23,24].

In this study, we present the first immunogenicity assessment of an ORFV-based vaccine combined with a licensed, adjuvanted recombinant protein vaccine approved for human use. We employed a simplified ORFV vector encoding only the spike antigen (ORFV-S) and evaluated its performance in homologous and heterologous prime–boost regimens with VidPrevtyn Beta. Using SARS-CoV-2 as a clinically relevant and immunologically well-characterized model, we systematically assessed humoral and cellular immune responses in mice to define the magnitude, quality, and cross-variant breadth of vaccine-induced immunity. Importantly, this work enables a comparison with earlier ORFV-based heterologous strategies involving adenoviral and inactivated vaccines, thereby providing new insights into platform-dependent immunological complementarity. Together, these findings highlight the potential of ORFV-containing mixed-platform vaccination strategies to enhance immune strength and diversity against rapidly evolving viral pathogens.

## 2. Materials and Methods

### 2.1. Ethics and Animals

All animal experiments were conducted in accordance with the recommendations of the Federation of European Laboratory Animal Science Associations (FELASA) and the guidelines of the regional authorities of Baden-Württemberg, Germany. Experimental procedures were performed at Synovo GmbH (Tübingen, Germany). All animal-related procedures, including handling, immunization, anesthesia, and euthanasia, were reviewed and approved by the competent regional authority, Regierungspräsidium Tübingen, under Project License No. 35/9185.81-7/SYN 12/20, approved on 1 January 2021. Female CD-1 mice (Charles River Laboratories, Sulzfeld, Germany) were used for all experiments. Animals were housed in accordance with German Animal Welfare Law and FELASA standards, with controlled environmental parameters and free access to food and water. Animal health and welfare were monitored daily throughout the study.

### 2.2. Study Design and Immunization

The ORFV-S encoding the full-length, prefusion-stabilized spike protein of the ancestral Wuhan SARS-CoV-2 strain was prepared as previously described [19,23]. The adjuvanted recombinant protein vaccine VidPrevtyn Beta (Sanofi–GSK) was purchased commercially and administered according to the manufacturer’s instructions.

At the start of the study, mice were 7–9 weeks of age. This outbred strain was selected to better reflect natural genetic diversity and to reduce strain-specific immunological bias, which is common practice in exploratory vaccine immunogenicity studies. Based on prior experience with ORFV-based and other vaccine platforms in murine immunogenicity models [19,23,24], animals were randomly allocated to experimental groups (*n* = 5–10 per group; total *n* = 50). Groups received either homologous or heterologous prime–boost vaccination regimens, with homologous vaccination serving as the control condition. The experimental unit was a single animal. Randomization was performed by assigning identification numbers to individual animals and distributing them across groups. Cage position, vaccination order, and sample processing order were balanced to minimize potential confounding effects.

Immunizations were administered intramuscularly under isoflurane anesthesia (3–4% in O_2_) on days 0 and 21. Animals received either 1/10 of the human dose of VidPrevtyn Beta or 1 × 10^6^ plaque-forming units (PFU) of ORFV-S, corresponding to approximately 1/100 of the intended human dose (1 × 10^8^ PFU). The intramuscular route was chosen to reflect the clinical use of both vaccine platforms and to enable a head-to-head comparison under clinically relevant conditions.

Peripheral blood samples were collected from the tail vein on days 14 and 28. On day 28, a subset of animals was euthanized by cardiac puncture under isoflurane anesthesia for spleen harvest, and splenocytes were isolated and cryopreserved according to standard protocols. At the final experimental time point (day 35), all remaining animals were euthanized by cardiac puncture under isoflurane anesthesia, and terminal blood samples were collected.

Predefined exclusion criteria included severe illness, technical failure during sample collection, or insufficient sample quality. No animals met these criteria, and no data points were excluded from the analysis. The exact number of animals included in each analysis is reported in the corresponding figure legends. All personnel involved in animal handling, experimental procedures, and outcome assessment were aware of group allocation.

### 2.3. Detection of Specific Serum IgG by ELISA

Spike-specific antibodies in mouse serum were quantified by indirect enzyme-linked immunosorbent assay (ELISA). High-binding 96-well plates (Nunc Maxisorp, Fisher Scientific, Schwerte, Germany) were coated overnight at 4 °C with 5 μg/mL of recombinant SARS-CoV-2 antigens in PBS (Thermo Fisher Scientific). The following proteins (all from Sino Biological, Beijing, China) were used for coating: full-length spike (Cat. No. 40589-V08B1), spike receptor-binding domain (RBD) with K417N mutation (Cat. No. 40592-V08H59), RBD(N501Y) (Cat. No. 40592-V08H82), and RBD(E484K) (Cat. No. 40592-V08H84). Plates were then blocked for 2 h at room temperature with 3% bovine serum albumin (BSA; Carl Roth, Karlsruhe, Germany) in PBS to prevent non-specific binding. Afterwards, 10-fold serial dilutions of serum samples were added to the wells and incubated for 1 h at room temperature. Antigen-bound antibodies were detected using horseradish peroxidase (HRP)-conjugated goat anti-mouse antibodies specific for total IgG (1:5000, Abcam, Cambridge, UK, ab6728), IgG1 (1:1000, Abcam, ab97240), or IgG2a (1:1000, Abcam, ab97245). Following 1 h incubation, plates were developed using TMB substrate (Cat. No. 421101, BioLegend) and the reaction was stopped after sufficient color development by adding stop solution (Cat. No. 423001, BioLegend). All samples and controls were run in duplicate. Absorbance was measured at 450 nm, and background values (blank wells) were subtracted from sample readings. Endpoint titers were determined by plotting the log_10_-transformed optical density (OD) values against the log_10_ of the serum dilution. A linear regression model was applied, and the endpoint titer was defined as the dilution at which the regression line of the sample intersected with the OD cut-off value of 0.1.

### 2.4. RBDCoV-ACE2 Measurements

RBDCoV-ACE2, a previously published multiplex ACE2 inhibition assay, analyses neutralizing antibody activity using ACE2 binding inhibition as a surrogate [25]. Neutralizing antibodies were analyzed against the SARS-CoV-2 WT, Beta, Delta, Omicron BA2 and XBB.1.5 variants. Samples were measured at a dilution factor of 1:400. In brief, RBD variant proteins were coupled to spectrally distinct populations of MagPlex beads (cat. no. MC100XX, Luminex, Austin, TX, USA) and then combined into a bead mix. Serum samples were diluted with assay buffer and then ACE2 buffer (300 ng/mL biotinylated ACE2, cat. no. 10108-H08H-B, Sino Biological), before being combined 1:1 with the bead mix in 96 well plates (cat. no. 3642, Corning, Corning, NY, USA). After incubation for 2 h at 21 °C, 750 rpm in a thermomixer, the beads were washed 3x in wash buffer using an automated microplate washer (Biotek 405TS, Winooski, VT, USA). Bound ACE2 was detected using 2 μg/mL Strep-PE (cat. no. SAPE-001, Moss, Pasadena, MD, USA) by incubating the bead-sample mix for a further 45 min. Following a further washing step, the beads were resuspended in 100 μL of wash buffer, shaken for 3 min at 1000 rpm and then measured on a FLEXMAP3D using the following settings: Timeout 80 s, Gate 7500–15,000, Reporter Gain: Standard PMT, 50 events. As controls, 150 ng/mL ACE2, blanks and 2 QC samples (all in duplicate) were included. ACE2 binding inhibition (%) was calculated as a percentage, with 100% indicating maximum ACE2 binding inhibition and 0% no ACE2 binding inhibition.

### 2.5. Intracellular Cytokine Staining (ICS)

Cryopreserved splenocytes were thawed, rested for 4 h at 37 °C in complete RPMI medium, and seeded into 96-well round-bottom plates (Greiner Bio-One, Frickenhausen, Germany) at a density of 2 × 10^6^ cells per well. Cells were re-stimulated with 0.5 μg/mL of SARS-CoV-2 full-length spike (PM-WCPV-S-1) peptide pools (JPT Peptide Technologies, Berlin, Germany) in the presence of 1 μg/mL anti-mouse CD28 (cat. no. 102116) and CD49d (cat. no. 103710) co-stimulatory antibodies (both BioLegend, San Diego, CA, USA). After 1 h of stimulation, Brefeldin A (10 μg/mL; Sigma-Aldrich, St. Louis, MO, USA), Monensin (cat. no. 420701, BioLegend), and anti-mouse CD107a antibody (cat. no. 121620, BioLegend) were added, and cells were incubated for an additional 14 h at 37 °C. Following stimulation, cells were blocked with TruStain FcX™ (anti-mouse CD16/32, cat. no. 101320, BioLegend) for 10 min at room temperature and subsequently stained for 30 min with a surface antibody cocktail containing anti-CD3ε (cat. no. 100312), CD4 (cat. no. 100531), CD8α (cat. no. 100730), CD62L (cat. no. 104430), and CD44 (cat. no. 103022), together with Zombie Aqua™ Fixable Viability Dye (cat. no. 423102, all BioLegend). Cells were then fixed and permeabilized using Fixation & Permeabilization Solution (BD Biosciences, Franklin Lakes, NJ, USA) for 30 min and stained intracellularly for cytokines with anti-mouse TNF-α (cat. no. 506344), IFN-γ (cat. no. 505835), IL-2 (cat. no. 503808), IL-4 (cat. no. 504104), and IL-17A (cat. no. 506941) antibodies (all BioLegend) for 30 min at room temperature. Samples were acquired on an Attune NxT flow cytometer (Thermo Fisher Scientific) and analyzed using FlowJo^®^ v10 software (BD Biosciences). Background responses from unstimulated control wells were subtracted from peptide-stimulated conditions. The gating strategy is shown in Supplementary Figure S1.

### 2.6. Statistical Analysis

Statistical analyses were performed using GraphPad Prism version 9 (GraphPad Software, San Diego, CA, USA). Normality of data distributions was assessed using the Shapiro–Wilk test. For normally distributed datasets, group comparisons were performed using an unpaired *t*-test with Welch’s correction for two-group comparisons, or Brown–Forsythe and Welch ANOVA followed by Dunnett’s T3 multiple-comparisons test for multi-group comparisons. For non-normally distributed datasets, comparisons were performed using the Mann–Whitney U test for two-group comparisons or the Kruskal–Wallis test followed by Dunn’s multiple-comparisons correction for multi-group analyses. For each assay and time point, statistical analyses were predefined to compare vaccinated groups pairwise with one another (homologous and heterologous regimens), as these comparisons directly address the objective of the study. The PBS group was included as a biological negative control to confirm baseline responses and assay specificity but was not included in the multiple-comparison statistical analyses. *p*-values < 0.05 were considered statistically significant and are indicated as follows: * *p* < 0.05; ** *p* < 0.01; *** *p* < 0.001; **** *p* < 0.0001. Data are presented as means ± standard deviation (SD), or geometric mean with geometric standard deviation (SD), as specified in the figure legends.

## 3. Results

**Figure 1:**
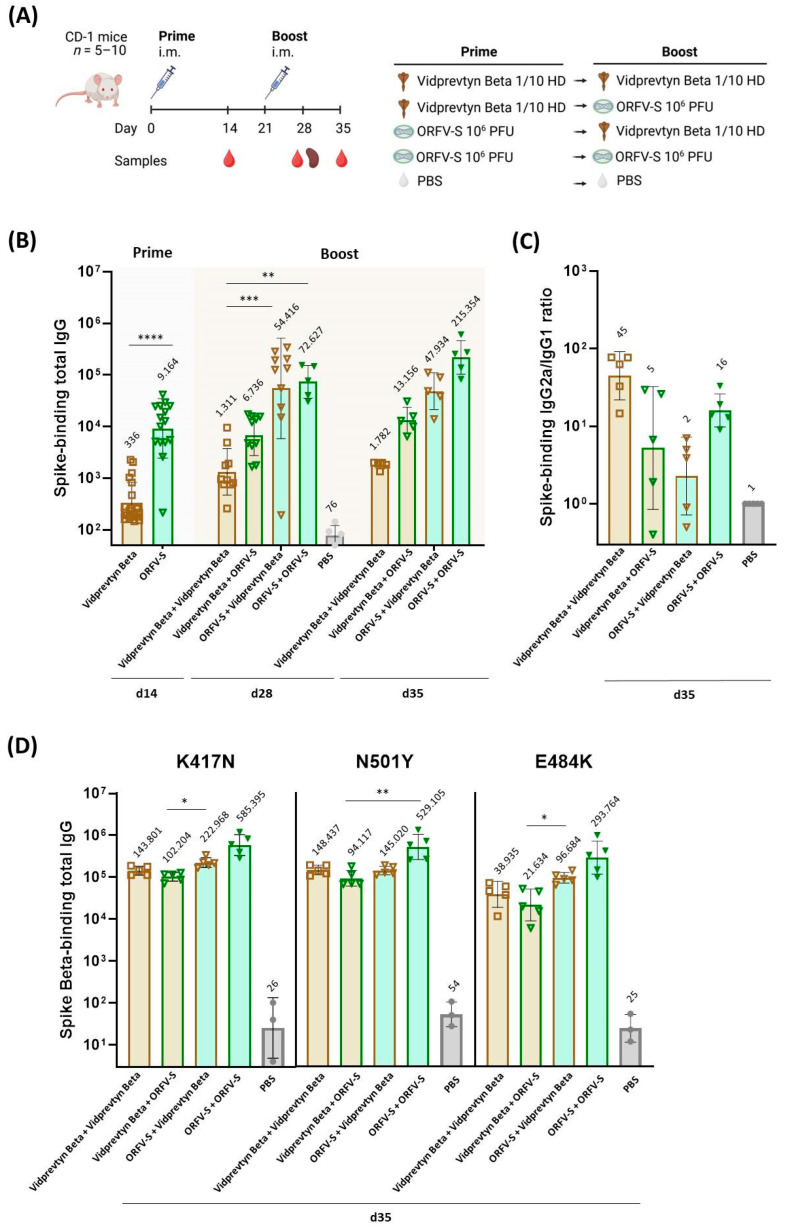
**Humoral immune responses following homologous and heterologous immunization with ORFV-S and VidPrevtyn Beta in CD-1 mice.** (**A**) Schematic overview of the experimental design, illustrating group allocation, vaccination schedule, and sample collection time points. (**B**) Endpoint titers of spike-specific total IgG against the ancestral Wuhan strain, measured by ELISA in serum collected two weeks after the prime immunization (day 14) and one and two weeks after the boost (days 28 and 35). (**C**) Ratio of IgG2a to IgG1 among Wuhan spike-specific antibodies on day 35, serving as an indicator of Th1/Th2 immune polarization. (**D**) Endpoint titers of spike-specific total IgG against the Beta (B.1.351) variant spike, containing the RBD mutations K417N, E484K, and N501Y, measured by ELISA in serum collected on day 35. Data are presented as geometric mean values ± geometric standard deviation (SD); geometric mean titers are indicated above the bars. Statistical analyses were performed as follows: for panel (**B**), day 14 data—Mann–Whitney U test, day 28—Kruskal–Wallis test followed by Dunn’s multiple-comparison correction, day 35—Brown–Forsythe and Welch ANOVA followed by Dunnett’s T3 multiple-comparison test; for panel (**D**), Brown–Forsythe and Welch ANOVA followed by Dunnett’s T3 multiple-comparison test. For each assay and time point, vaccination regimens were compared pairwise with one another. Statistical comparisons were limited to vaccination regimens; the PBS group served as a biological control. Statistical significance is indicated as follows: * *p* < 0.05; ** *p* < 0.01; *** *p* < 0.001; **** *p* < 0.0001.

**Figure 2:**
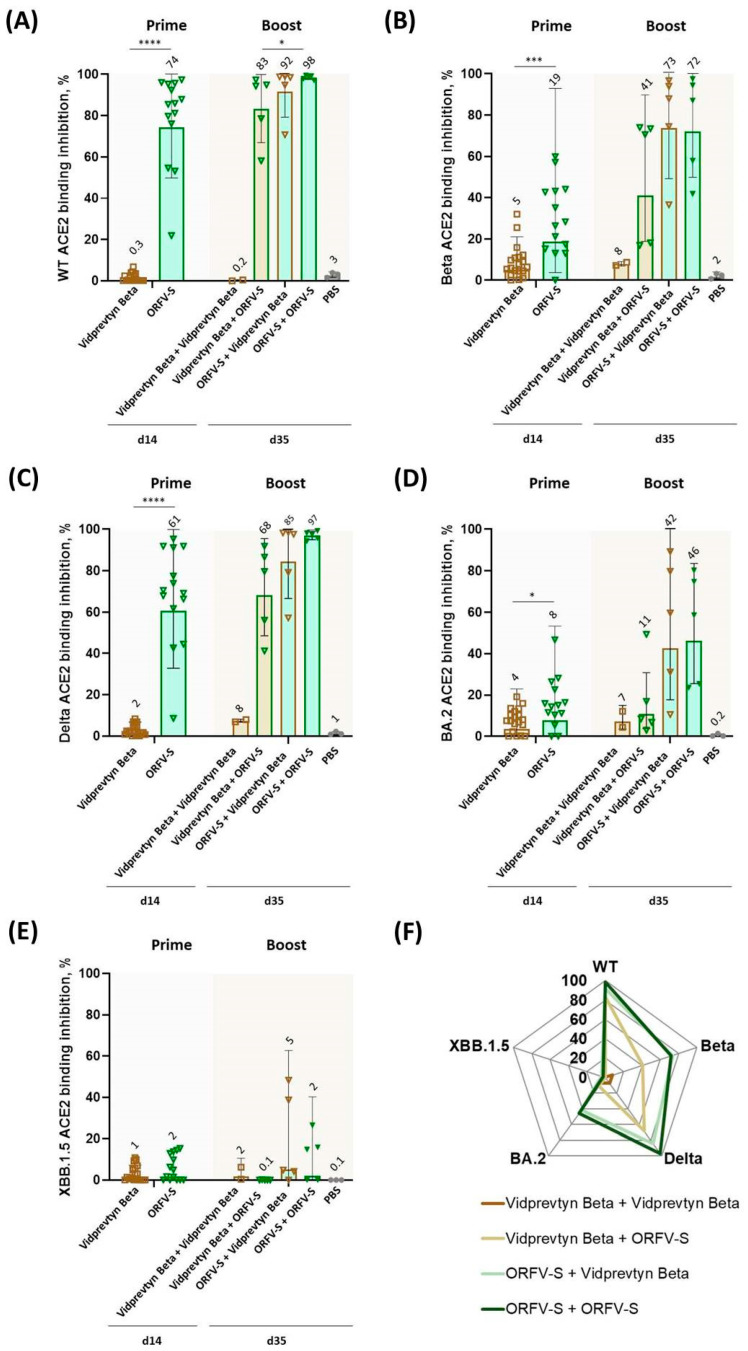
**ACE2 binding inhibition against SARS-CoV-2 variants of concern (VoC) following homologous and heterologous immunization with ORFV-S and VidPrevtyn Beta, assessed by the RBDCoV-ACE2 assay.** ACE2 binding inhibition (%) in serum was measured on day 14 and day 35 against the RBDs of (**A**) wild-type (WT), (**B**) Beta, (**C**) Delta, (**D**) Omicron BA.2, and (**E**) Omicron XBB.1.5. Data are shown as geometric mean values ± geometric standard deviation (SD); geometric mean values are indicated above the bars. (**F**) Radar chart displaying geometric mean ACE2 binding inhibition (%) on day 35 across all five variants. All samples were tested at a fixed serum dilution of 1:400. Responses < 0.1% were set to 0.1% for visualization; values > 20% were considered indicative of a positive response. Statistical analyses were performed as follows: for panels (**A**,**B**), day 14—Mann–Whitney U test, day 35—Kruskal–Wallis test followed by Dunn’s multiple-comparison correction; for panel (**C**), day 14—Mann–Whitney U test, day 35—Brown–Forsythe and Welch ANOVA followed by Dunnett’s T3 multiple-comparison test; for panel (**D**), day 14—unpaired t test with Welch’s correction, day 35 data—Brown–Forsythe and Welch ANOVA followed by Dunnett’s T3 multiple-comparison test; for panel (**E**), day 14—Mann–Whitney U test, day 35—Kruskal–Wallis test followed by Dunn’s multiple-comparison correction. Vaccination regimens were compared pairwise with one another. Statistical comparisons were limited to vaccination regimens; the PBS group served as a biological control. Statistical significance is indicated as follows: * *p* < 0.05; *** *p* < 0.001; **** *p* < 0.0001.

Mice were immunized with homologous or heterologous prime–boost regimens combining the replication-deficient ORFV-S vector and the adjuvanted recombinant protein vaccine VidPrevtyn Beta. Humoral and cellular immune responses were analyzed two weeks after each dose to assess the magnitude, quality, and cross-variant breadth of vaccine-induced immunity (Figure 1 and Figure 2). Outcome measures included spike-specific serum IgG endpoint titers, IgG subclass distributions (IgG2a/IgG1 ratios), ACE2 binding inhibition as a surrogate for neutralizing antibody activity, and the frequencies of spike-specific CD4^+^ and CD8^+^ T cells producing IFN-γ, TNF, IL-2, or expressing CD107a. All statistical comparisons presented in the figures were performed according to the predefined analysis plan described in the Methods section, using parametric or non-parametric tests as appropriate.

### 3.1. Spike-Specific IgG Responses to the Ancestral Wuhan Strain

Two weeks after prime immunization, VidPrevtyn Beta (1/10 of the human dose) elicited detectable spike-specific IgG antibodies cross-reactive to the ancestral Wuhan strain, although titers were significantly lower than those induced by a single dose of ORFV-S at 10^6^ PFU (*p* < 0.0001) (Figure 1A,B). One week after the boost, homologous ORFV-S immunization produced significantly higher antibody titers than homologous VidPrevtyn Beta (*p* < 0.01) (Figure 1B). The heterologous regimen in which ORFV-S priming was followed by VidPrevtyn Beta boosting generated markedly increased binding antibody levels, significantly exceeding those elicited by homologous VidPrevtyn Beta immunization (*p* < 0.001) (Figure 1B). Antibody titers in this ORFV-S → VidPrevtyn Beta group approached the high levels observed after homologous ORFV-S vaccination at the same time point, suggesting that ORFV-S priming establishes a robust humoral baseline that can be efficiently amplified by subsequent protein boosting.

### 3.2. IgG Subclass Profiles and Th1/Th2 Bias

To assess the quality of the humoral response, IgG subclass distributions were analyzed in sera collected two weeks after the second immunization. The IgG2a/IgG1 ratio was used as an indicator of Th1 versus Th2 immune polarization. All vaccination regimens exhibited a predominance of IgG2a over IgG1, consistent with a Th1-biased humoral response across groups (Figure 1C).

### 3.3. Spike-Specific IgG Responses to the Beta Variant

To assess antibody cross-reactivity, spike-specific IgG titers against the Beta (B.1.351) variant, bearing the RBD mutations K417N, N501Y and E484K, were determined by ELISA two weeks after the second immunization. Both homologous ORFV-S and homologous VidPrevtyn Beta regimens elicited robust IgG responses against the Beta spike (Figure 1D). In heterologous regimens, priming with ORFV-S followed by VidPrevtyn Beta boosting significantly enhanced antibody titers compared with the reverse sequence (*p* < 0.05), yielding 1.6- and 2.5-fold higher responses against the K417N and E484K mutations, respectively (Figure 1D).

### 3.4. ACE2 Binding Inhibition Against SARS-CoV-2 Variants of Concern

To evaluate the functional activity of vaccine-induced antibodies, sera were analyzed using the RBDCoV-ACE2 inhibition assay. Two weeks after a single dose, ORFV-S induced statistically strongest ACE2 binding inhibition against the ancestral (wild-type, WT) and Delta variants (*p* < 0.0001), with moderate activity against Beta (*p* < 0.001) and lower against BA.2 (*p* < 0.05) variants as compared to those induced by a single dose of VidPrevtyn Beta (Figure 2A–D).

Following booster immunization, both heterologous regimens and the homologous ORFV-S schedule elicited potent ACE2 binding inhibition across the WT, Beta, and Delta variants (Figure 2A–C). As expected, ACE2 inhibition against Omicron-related variants was reduced across all groups (Figure 2D,E). The ACE2 binding inhibition profiles observed across SARS-CoV-2 variants following immunization with ORFV-based regimens, alone or in combination with an adjuvanted recombinant protein vaccine, are summarized in Figure 2F.

### 3.5. Spike-Specific CD4^+^ and CD8^+^ T Cell Responses

To characterize vaccine-induced cellular immunity, we quantified spike-specific CD4^+^ and CD8^+^ T cells in splenocytes by intracellular cytokine staining (ICS) after ex vivo stimulation with peptide pools from the ancestral Wuhan spike. Despite antibody escape, overall T cell recognition of the Beta (B.1.351) variant remains largely preserved, as >90% of spike-specific epitopes are conserved between the ancestral and Beta strains [26,27].

**Figure 3:**
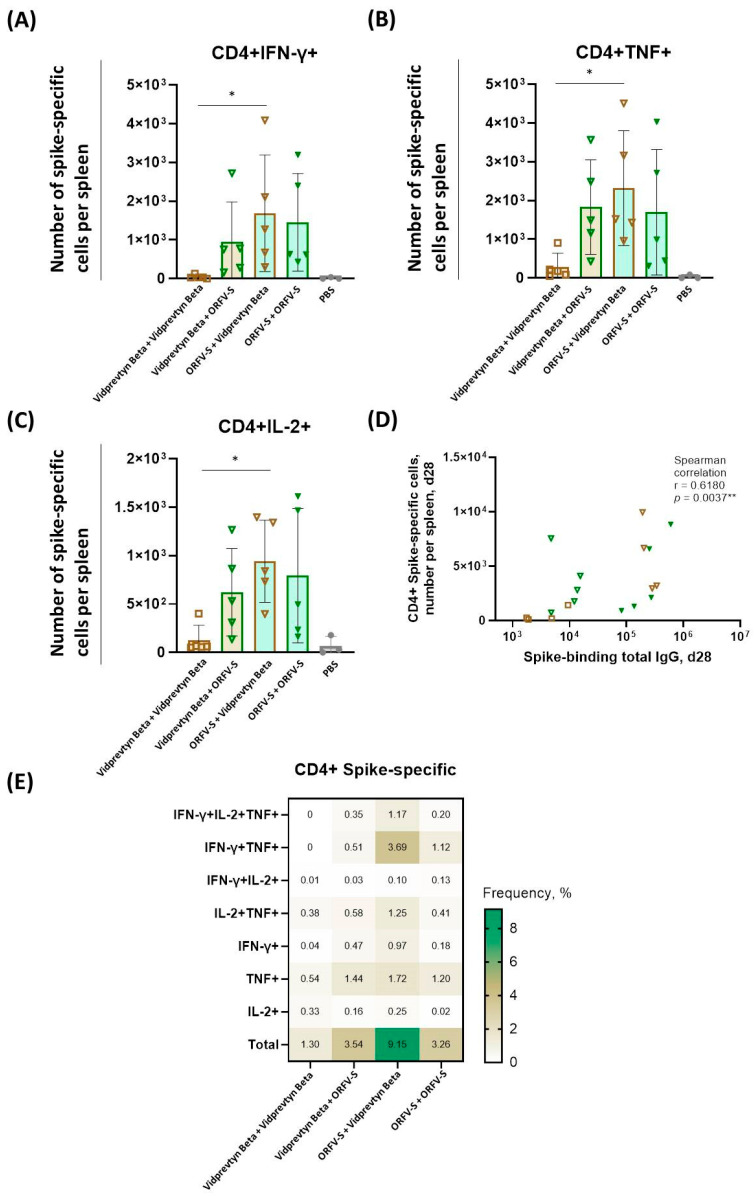
**Spike-specific CD4^+^ T cell responses induced by homologous and heterologous vaccination with ORFV-S and VidPrevtyn Beta in CD-1 mice.** (**A**–**C**) Numbers of CD4^+^ T cells producing (**A**) IFN-γ, (**B**) TNF, and (**C**) IL-2 per spleen following ex vivo stimulation with peptide pools spanning the SARS-CoV-2 spike protein (ancestral Wuhan strain), measured by intracellular cytokine staining (ICS) on day 28. Bar heights represent mean ± SD. Statistical analyses for panels (**A**–**C**) were performed using the Kruskal–Wallis test followed by Dunn’s multiple-comparison correction. All vaccination regimens were compared pairwise with one another. Statistical comparisons were limited to vaccination regimens; the PBS group served as a biological control. (**D**) Correlation between the numbers of spike-specific CD4^+^ T cells per spleen and total spike-specific IgG endpoint titers in serum on day 28. (**E**) Heatmap showing the mean frequencies of polyfunctional spike-specific CD4^+^ T cells per group, measured in splenocytes on day 28. Statistical significance is indicated as follows: * *p* < 0.05, ** *p* < 0.01.

Among tested regimens, homologous VidPrevtyn Beta vaccination induced the lowest CD4^+^ T cell frequencies (Figure 3A–C). Incorporating ORFV-S as a booster increased CD4^+^ responses, approaching those elicited by homologous ORFV-S vaccination. Conversely, priming with ORFV-S followed by VidPrevtyn Beta boosting resulted in significantly higher CD4^+^ T cell frequencies compared with homologous VidPrevtyn Beta vaccination (*p* < 0.05), indicating a robust helper T cell profile.

Spike-specific Th1 cytokine levels correlated positively with IgG titers (Figure 3D), indicating coordinated humoral and cellular immunity. Analysis of T cell polyfunctionality revealed that the ORFV-S → VidPrevtyn Beta regimen generated higher frequency of multifunctional CD4^+^ cells (IFN-γ^+^/TNF^+^/IL-2^+^) as compared to homologous VidPrevtyn Beta vaccination (*p* < 0.01) (Figure 3E, Supplementary Figure S2).

**Figure 4:**
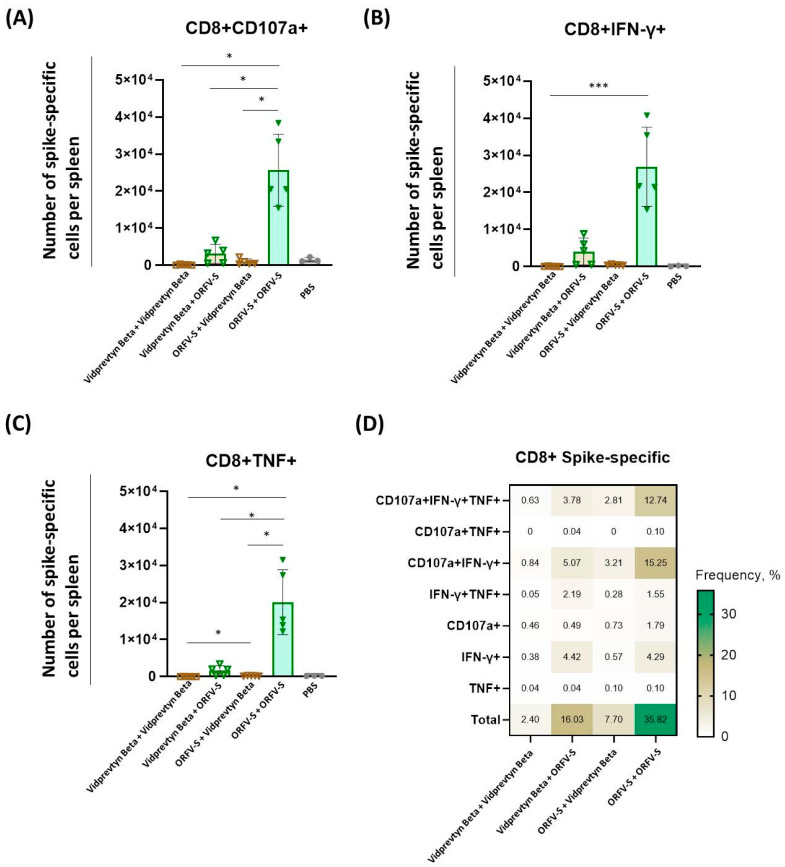
**Spike-specific CD8^+^ T cell responses induced by homologous and heterologous vaccination with ORFV-S and VidPrevtyn Beta in CD-1 mice.** (**A**–**C**) Numbers of CD8^+^ T cells producing (**A**) CD107a, (**B**) IFN-γ, and (**C**) TNF per spleen following ex vivo stimulation with peptide pools spanning the SARS-CoV-2 spike protein (ancestral Wuhan strain), measured by intracellular cytokine staining (ICS) on day 28. Bar heights represent mean ± SD. Statistical analyses were performed as follows: for panels (**A**,**C**), Brown–Forsythe and Welch ANOVA followed by Dunnett’s T3 multiple-comparison test; for panel (**B**), Kruskal–Wallis test followed by Dunn’s multiple-comparison correction. All vaccination regimens were compared pairwise with one another. Statistical comparisons were limited to vaccination regimens; the PBS group served as a biological control. (**D**) Heatmap showing the mean frequencies of polyfunctional spike-specific CD8^+^ T cells per group, measured in splenocytes on day 28. Statistical significance is indicated as follows: * *p* < 0.05; *** *p* < 0.001.

VidPrevtyn Beta alone also induced low CD8^+^ T cell activation (Figure 4A–C). ORFV-S priming followed by VidPrevtyn Beta boosting did not further enhance CD107a^+^ or IFN-γ^+^ CD8^+^ responses, and no statistically significant differences were observed compared with the reverse sequence (VidPrevtyn Beta → ORFV-S). Homologous ORFV-S vaccination elicited the strongest CD107a^+^ and TNF^+^ CD8^+^ T cell responses overall (*p* < 0.05) (Figure 4A,C).

Assessment of CD8^+^ T cell polyfunctionality demonstrated that homologous ORFV-S vaccination resulted in significantly higher percentage of polyfunctional CD8^+^ T cells (CD107a^+^/IFN-γ^+^/TNF^+^) as compared to homologous VidPrevtyn Beta regimen (*p* < 0.01) (Figure 4D, Supplementary Figure S2). Together, these data highlight the strong T cell activating capacity of the ORFV vector in mixed-platform regimens.

## 4. Discussion

In this study, we investigated whether an ORFV-based spike vaccine can be effectively combined with a licensed, adjuvanted recombinant protein vaccine in a heterologous prime–boost regimen, using SARS-CoV-2 as a clinically relevant model. This is the first time to evaluate the immunogenicity of an ORFV-based vector in combination with a commercially approved recombinant protein vaccine, thereby extending previous ORFV heterologous strategies to a new and clinically relevant vaccine class.

Previous ORFV-based heterologous prime–boost studies demonstrated synergistic immune enhancement when ORFV vectors were combined with adenoviral [24] or inactivated SARS-CoV-2 vaccines [23]. While these studies established ORFV as a versatile vector platform, they did not examine whether ORFV could similarly enhance immunity induced by protein-based vaccines, which differ fundamentally in their underlying immunological properties.

Recombinant protein vaccines are widely used in human immunization programs due to their excellent safety record and scalable manufacturing. The ORFV has emerged as a promising vaccine vector, characterized by strong innate activation via cGAS-STING pathway, low pre-existing immunity, and the ability to elicit robust cellular and humoral responses [17,18,19,23,28]. Although heterologous prime–boost regimens combining protein vaccines with viral vectors have previously demonstrated immunological advantages for HIV [29], malaria [30], RSV [31], and SARS-CoV-2 [32], the potential of ORFV in such mixed-platform regimens had not yet been assessed. We therefore explored whether pairing ORFV-S with VidPrevtyn Beta could augment immune responses against SARS-CoV-2.

Our findings show that ORFV-S alone induced potent and broad SARS-CoV-2–specific immune responses. Homologous ORFV-S vaccination efficiently primed and boosted cross-reactive antibody responses extending from the ancestral Wuhan spike to the immune-evasive Beta (B.1.351) variant harboring the RBD mutations K417N, E484K, and N501Y. Homologous ORFV-S immunization generated the highest overall IgG titers and the broadest ACE2-binding inhibition across variants of concern, highlighting the intrinsic potency and breadth of this poxvirus vector platform.

In heterologous regimens, ORFV-S acted as an effective priming component. ORFV-S priming followed by VidPrevtyn Beta boosting markedly enhanced antibody magnitude and cross-variant reactivity compared with homologous protein vaccination, confirming the benefit of vector priming in mixed-platform schedules. Similar advantages of vector–protein combinations have been reported for an Ad5 prime–protein boost COVID-19 regimen [33]. Although the heterologous ORFV-S → VidPrevtyn Beta regimen did not exceed the antibody levels achieved by homologous ORFV-S immunization, it substantially improved humoral responses relative to protein-only vaccination. These findings support the value of incorporating ORFV-S into heterologous regimens, particularly in settings where vector dose-sparing or broadening of protein-induced immunity is desirable.

The cellular data further emphasize the strong T cell–stimulating capacity of the ORFV vector. Both homologous and heterologous regimens containing ORFV-S elicited substantial CD4^+^ and CD8^+^ T cell responses, whereas VidPrevtyn Beta alone induced only modest T cell activity. ORFV-S boosting effectively enhanced CD8^+^ T cells following protein priming, an important feature given the limited cytotoxic T cell induction by protein vaccines, which lack intracellular antigen expression [33]. CD4^+^ T cell responses mirrored the humoral response data: the ORFV-S → VidPrevtyn Beta regimen induced the highest frequencies of polyfunctional CD4^+^ helper T cells, consistent with a robust helper T cell profile.

The observed cellular immune profiles may provide a mechanistic framework for the magnitude and breadth of the humoral responses induced by ORFV-S-containing regimens. The predominance of Th1-biased CD4^+^ T cell responses, characterized by IFN-γ, TNF, and IL-2 production, is known to support efficient germinal center formation, antibody affinity maturation, and class switching toward IgG2a-dominated responses, consistent with the IgG subclass profiles detected here [34]. Such Th1-oriented helper responses may therefore contribute to the enhanced antibody magnitude and cross-variant reactivity observed following ORFV-S priming, particularly in the ORFV-S → VidPrevtyn Beta regimen. Moreover, the enrichment of polyfunctional CD4^+^ T cells producing several Th1 cytokines is considered a hallmark of T cell help and has been associated with durable and broadly protective humoral immunity in vaccine settings [35,36,37]. The positive correlation between Th1 cytokine–producing CD4^+^ T cells and spike-specific IgG titers observed in this study further supports a coordinated interplay between cellular and humoral immunity. These data suggest that ORFV-S not only increases antibody levels but may also qualitatively shape the helper T cell environment required for the generation of potent and cross-reactive antibody responses.

Together, these findings position ORFV-S as a potent vaccine component capable of driving broad, Th1-oriented humoral and cellular immunity. When paired with a recombinant protein vaccine, ORFV-S enhanced antibody magnitude and cross-variant breadth beyond that achieved by homologous protein vaccination and effectively complemented the protein platform’s limited T cell induction.

Importantly, these findings have direct translational relevance. The ability of ORFV-S to induce robust humoral and cellular immunity at sub-dose levels highlights its potential for dose-sparing strategies, which are particularly valuable in pandemic settings where manufacturing capacity and rapid global deployment are limiting factors. From a regulatory and practical perspective, the use of a licensed recombinant protein vaccine such as VidPrevtyn Beta as a booster offers clear advantages, leveraging an established safety profile, existing manufacturing infrastructure, and familiar regulatory pathways. This mixed-platform strategy is therefore readily adaptable to current booster campaigns, including those targeting emerging variants, and could be implemented without the need for entirely new vaccine authorizations. Moreover, given that large proportions of the global population have already been primed with protein-, adenoviral-, or inactivated-vaccine platforms, ORFV-based vectors may represent a flexible boosting option capable of enhancing both antibody breadth and T cell immunity in previously vaccinated individuals. In this context, our data support the clinical exploration of ORFV-based heterologous boosting strategies as a means to strengthen and broaden immunity in the face of ongoing viral evolution.

Our study has several limitations. Although robust humoral and cellular immune responses were observed, no in vivo viral challenge experiments were performed; therefore, direct evidence of protective efficacy against SARS-CoV-2 infection is lacking, particularly given that no robust immune correlate of protection has been established. The immune responses were analyzed at early time points after vaccination, and long-term durability as well as memory B- and T-cell responses were not assessed. All experiments were conducted in a murine model, which, while suitable for comparative immunogenicity studies, may not fully reflect human immune responses. Finally, immunity was evaluated primarily in the systemic compartment, and mucosal immune responses—particularly relevant for respiratory pathogens such as SARS-CoV-2—were not examined. Addressing these aspects in future studies will be important to further define the protective and translational potential of ORFV-based heterologous vaccination strategies.

Mechanistic investigations exploring innate sensing and antigen presentation pathways may further elucidate the sequence-dependent effects observed here. Nevertheless, our results provide a compelling rationale for clinical exploration of ORFV-based heterologous prime–boost strategies, particularly in pandemic preparedness contexts where broad, durable, and T cell-supported immunity is critical.

## 5. Conclusions

The replication-deficient ORFV-S vector induces broad and potent Th1-oriented humoral and cellular immunity. In heterologous prime–boost combinations with adjuvanted recombinant protein vaccines, ORFV-S enhances antibody magnitude and cross-variant breadth beyond homologous protein vaccination. These findings support the use of ORFV as a versatile component in mixed-platform vaccine strategies against rapidly evolving viral pathogens.

## Data Availability

The original contributions presented in the study are included in the article. Further inquiries can be directed to the corresponding author.

## Supplementary Materials

The following supporting information can be downloaded at: https://www.mdpi.com/article/10.3390/vaccines14010064/s1, Supplementary Figure S1. Representative gating strategy for intracellular cytokine staining analysis of spike-specific CD4⁺ and CD8⁺ T cells following ex vivo peptide stimulation; Supplementary Figure S2. Polyfunctional spike-specific CD4⁺ (upper panels) and CD8⁺ (lower panels) T cell responses induced by homologous and heterologous vaccination with ORFV-S and VidPrevtyn Beta in CD-1 mice.
