# Supplementary material for: Broadening SARS-CoV-2 Immunity by Combining ORFV and Protein-Based Vaccines
Source: Vaccines (Basel). 2026 Jan 4;14(1):64. doi: 10.3390/vaccines14010064 (PMC12846268; doi:10.3390/vaccines14010064)

Supplementary Figure S1. Representative gating strategy for intracellular cytokine staining analysis of spike-specific CD4⁺ and CD8⁺ T cells following *ex vivo* peptide stimulation.

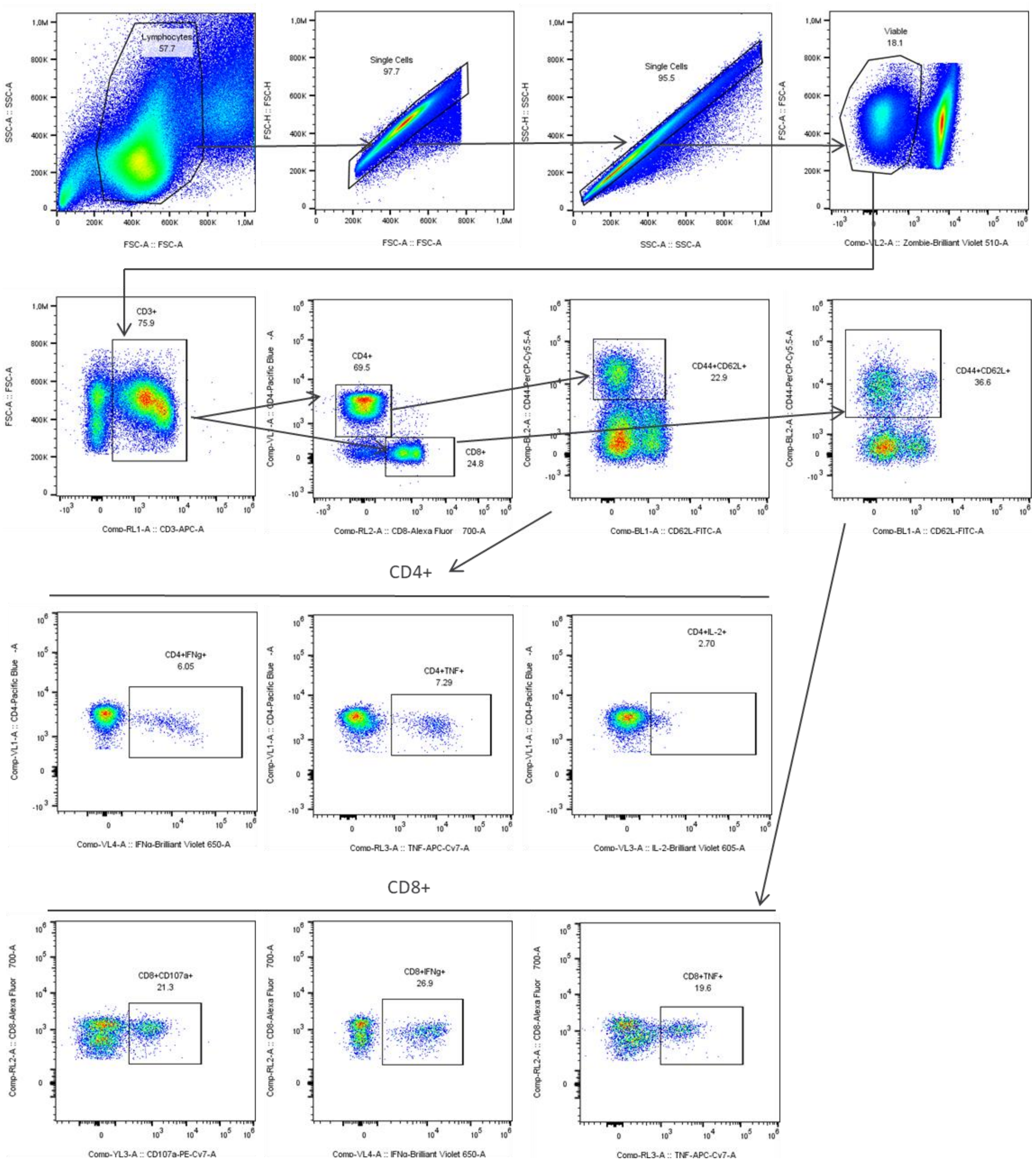

Supplementary Figure S2. Polyfunctional spike-specific CD4^{+} (upper panels) and CD8^{+} (lower panels) T cell responses induced by homologous and heterologous vaccination with ORFV-S and VidPrevtyn Beta in CD-1 mice. Splenocytes were collected on day 28 and re-stimulated *ex vivo* with peptide pools spanning the SARS-CoV-2 spike protein (ancestral Wuhan strain). Polyfunctional T cells were identified by intracellular cytokine staining (ICS) based on the simultaneous expression of the markers. Data are presented as mean ± standard deviation (SD). Statistical analyses were performed using the Kruskal–Wallis test followed by Dunn's multiple-comparison correction. Statistical comparisons were limited to vaccination regimens; the PBS group served as a biological control. Statistical significance is indicated as follows: *p < 0.05; **p < 0.01.

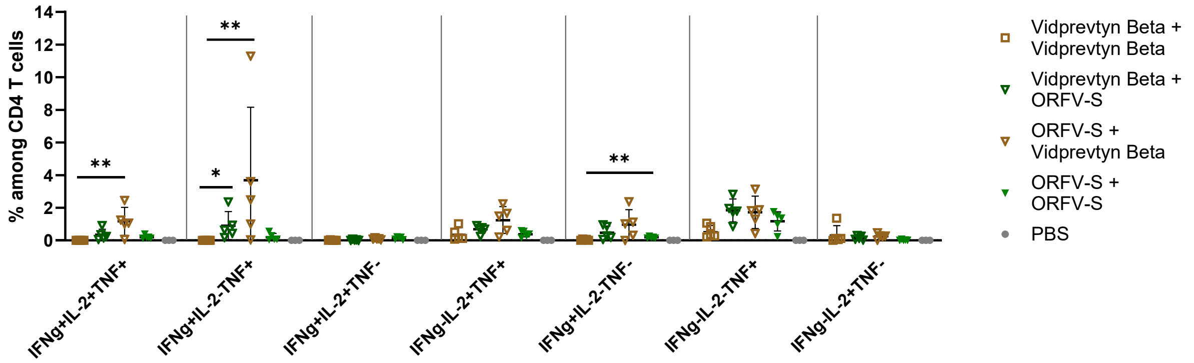

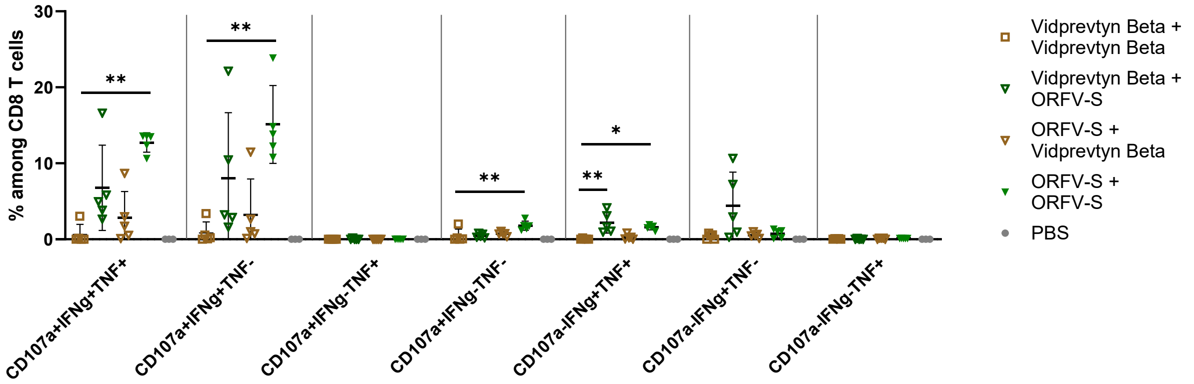

Supplement: Supplementary file 1 [file vaccines-14-00064-s001.zip › vaccines-4025881-supplementary.pdf]
